# Limiting lifetime inheritances and gifts

**DOI:** 10.1177/1470594X241277959

**Published:** 2024-09-05

**Authors:** Ingrid Robeyns

**Affiliations:** Department of Philosophy, 100399 Faculty of Humanities, Utrecht University, Utrecht, the Netherlands

**Keywords:** inheritance, bequest, gifts, accessions, tax, fiscal policies, limitarianism, citizen's stake

## Abstract

This paper provides a defence of a lifetime limit on how much a person can receive in the form of gifts and inheritances, that is, a person's *accessions*. I argue that any accessions above that limit should be taxed at a rate of 100%. The analysis shows how this proposal can bring into equilibrium the core values and other goals at stake in the design of taxation on inheritances and gifts. To counter the low level of support for inheritance taxation in general, the analysis must include a proposal concerning what will be done with the tax revenues. I propose turning the accession tax revenues into an unconditional citizen's stake that is granted to young adults. Such an institutional design would create additional value by reducing unfair inequalities between age groups.

## The accessions cap proposal

Globally, there is a wide diversity in inheritance and bequest taxation legislation. Several countries have abolished inheritance and gift taxation completely; other countries continue to hold onto significant taxation rates, though these are often combined with generous deductions, in particular for assets such as family firms or for particular groups of heirs (OECD, 2021). Continued discussion about gift and inheritance taxation is still very important given the staggering increase in wealth inequalities alongside a status quo in which income and consumption tend to be the principal tax bases, even though the wealthiest tend not to be burdened by them. If gifts and inheritances are not taxed according to tax rates that are fair, massive levels of unjust transfers of wealth will take place over the coming decades. If everyone received the same level of inheritances and gifts, perhaps we would not have to worry. As Atkinson (2015: 170) points out, “there is nothing intrinsically wrong with inheritance. The problem is that inheritance is highly unequal.” Thus, we do have reasons to worry about this problem and investigating taxation designs that we should consider to endorse.

In this paper, I argue that if we want to provide a normatively justified real-world design for inheritance and gift taxations, we should conduct a comprehensive normative audit as our method of analysis. We should also be very explicit about the status of our design, namely, it is a *proposal* to be offered to citizens for debate (and subsequent endorsement, modification, or rejection) in their democratic deliberations. The proposal that I defend is the Accessions Cap Proposal:

*The Accessions Cap Proposal:* the government imposes a lifetime limit on what each person can receive in terms of inheritances and gifts. Once that limit is reached, the government implements a 100% tax rate on any further inheritances or gifts received. The tax revenue thereby generated is used to finance a citizen's stake.

This proposal combines design features which have previously been defended. The first feature is that when regulating the taxation of wealth transfers (whether *inter vivos* or upon death), the tax should be payable by the *recipients* of the wealth. Given space limitations, I will not defend this aspect of the Accessions Cap Proposal explicitly, as I take it to be the least controversial one and also because others have provided strong arguments in its favor (Atkinson, 2015; Duff, 1993; Fleischer, 2016; Mill, 1848; Rudick, 1945).

Second, I will argue for a combined lifetime tax on inheritance and gifts. Such a tax has been coined an “accession tax” by Rudick (1945). Rudick does not specify what rates would be optimal, but nothing in his proposal suggests he thought that the maximal marginal tax rate on accessions would be 100%. Choosing to combine inheritances and gifts is also an uncontroversial design feature, and many scholars writing about inheritance tax have used and continue to use “inheritance” as shorthand for “inheritances and gifts” (e.g., Pedersen, 2018).

This brings us to the third feature of the Accessions Cap Proposal, namely a 100% marginal tax rate above the cap, or limit. Each person would be allowed to receive gifts and inheritances until they reach the accession cap, after which further donations to that person would be taxed at a rate of 100%.^1^

The first three elements of the Accessions Cap Proposal have been defended before by Haslett (1997), who acknowledges that Mill (1848) also advocates a lifetime quota. However, Mill has often been interpreted as more moderate in his policy recommendations, since he conceives of the quota as the limit at which the tax-free exemption ends and advocates *progressive* taxation (rather than confiscatory taxation) above that limit (Cappelen and Pedersen, 2018). Instead, following Haslett, I will argue for a lifetime quota of inheritances and gifts and 100% taxation above that cap, because this reflects a more plausible balance of the different values and principles at stake. Clearly, this drastically increases the feasibility objection (Cappelen and Pedersen, 2018: 323), which is an objection that Haslett does not address. However, if we are aiming for a real-word analysis and institutional design, we should take the feasibility concerns seriously, and that is why we need to add a fourth feature to the Accessions Cap Proposal.

The fourth and final part of the Accessions Cap Proposal is a form of *hypothecation*—the earmarking of specific tax revenues for specific policies (Halliday, 2018: 204). The revenue generated by an accessions tax should be turned into a citizen's stake—a capital grant endowed to all citizens when they reach adulthood (Ackerman and Alstott, 1999; Dowding et al., 2003; Paxton and White, 2006). One could argue for an accession cap without specifying what will be done with the tax revenue, but for reasons of feasibility a stake is needed; this will raise democratic support for an accession tax. The proposal to fund a citizen's stake with the tax revenue from inheritance taxation goes back to Paine (1796), who argued that such a stake was grounded in the fact that with the onset of agriculture, citizens had lost their common right to land and thus had to be compensated for that loss by those who were able to acquire wealth by cultivating that land. He argued that the loss of common ownership of land would justify an inheritance tax rate of 10%. More recently, Le Grand and Nissan (2003) have argued for a citizen's stake of £10,000, funded by progressive inheritance taxation. Thomas Paine proposes a flat inheritance tax rate of 10%; Le Grand and Nissan review several scenarios with average tax rates of 14% to 25%. Thus, while the Accessions Cap Proposal shares with these authors’ proposals the hypothecation of inheritance taxation creating a citizens’ income, the accession tax rates proposed in this paper are very different. This is because the aim of the method I use to argue for a cap on accessions is to be as comprehensive as possible when evaluating the effects of gifts and inheritances on public values and principles.

How does the Accessions Cap Proposal differ from what Haslett proposes? He endorses what he calls a “productivity ideal of distributive justice,” which entails the following view: “to all people according to the productivity of their labor, or of the property they have acquired in return for the productivity of their labor” (Haslett, 1997: 136). Since inheritances and gifts do not reflect any such productivity, and if the only value that counts is this productivity-related ideal of distributive justice, there should not be any inheritances or gifts. This is precisely what contemporary political philosophers, following Mill, say when they point out that no one can be said to deserve an inheritance. Haslett presents his proposal for a lifetime quota as a compromise between distributive justice, freedom, and equality.

The Accessions Cap Proposal modifies and extends the arguments of Haslett in two ways. The first way arises from the fact that Haslett does not consider all relevant values and principles. In contrast, I conducted a comprehensive normative audit, which does not prioritize desert (or the productivity ideal) over other values that citizens might have reasons to find morally relevant when thinking about inheritance and gift taxation. Both Mill and Haslett placed distributive justice at the center of their analysis, but we need to broaden that perspective (Schmidt am Busch, 2023). The method I use is eminently suitable for the task of broadening our normative analysis beyond a focus only on justice. In addition, in the method that I use, feasibility constraints take central stage. A crucial feasibility constraint on accessions taxation is the lack of democratic support. In contrast to Haslett, who ignores most feasibility challenges, I will address them when unfolding my analysis and defending the Accessions Cap Proposal.

## Comprehensive normative audit

The Accessions Cap Proposal is meant to be a contribution to a democratic debate about the current or a nearby world. This implies that we need a method suitable for real-world normative political philosophy. I call the method that I will use “comprehensive normative audit.”^2^ This method is very similar to the methods that Jonathan Wolff (2015, 2018) has discussed and is congruent with a range of methodological positions of authors who are interested in developing methods for the analysis of concrete normative issues (e.g., Goodin et al., 1999; Hamlin and Stemplowska, 2012). Comprehensive normative audit is not a new method; instead, I try to make explicit a method that is implicitly used in the literature on real world political philosophy.

Citizens who deliberate about the institutions and policies they want generally use the language of *value pluralism*—acknowledging the existence of many values that are relevant to the design of institutions.^3^ However, citizens weigh the relative importance of those values differently. Often, normative theories give sole priority, or lexical priority, to one value, e.g., to freedom, distributive justice, or welfare. Philosophers typically analyze inheritance and gift-giving by starting from a particular normative position or a theory such as classical liberalism (Haslett, 1986), luck egalitarianism (Lazenby, 2010), or Ronald Dworkin's theory of equality of resources (Halliday, 2016). As Wolff points out, for such theory-based analyses to be helpful for public deliberation, those who adopt the theories and the outcomes must accept the dogmatic aspects of the theories as well, such as the lexical priority given to a certain value or principles, or other normative assumptions, which may also be deeply contested. A comprehensive normative audit analyses the relationship between all *relevant values* and the institutional design; from these analyses, *pro tanto* arguments for a certain design feature will emerge. Citizens will have to do their own balancing of values and find an institutional design that best reflects their weighing of those values.

When a philosopher is adopting this methodological and meta-theoretical stance, they have two tasks. First, they need to analyze the relationship(s) between the value and the societal issue, which will lead to *pro tanto* reasons for specific design features. Second, the philosopher can *either* lay out a range of institutional designs and explain how weighing the values differently favors one or more institutional designs, *or* they can embrace the prescriptive option, argue for a specific institutional design and discuss how it corresponds to those values and how it weighs them. Much of the normative policy-oriented literature does the latter, but it seldom takes a wide enough range of relevant values into account. Very few analyses aim to be as comprehensive as possible.

There is a deeper meta-theoretical concern lurking here. Covering many values in a single paper may raise the concern that any analysis will lack the precision and detail of more narrowly focussed or incremental attempts at making progress. However, this conclusion does not follow. The first reason for this is that a comprehensive normative audit *synthesizes* other analyses that only look at one or a few values in more depth. The audit synthesizes the depth provided elsewhere. The second reason is that action-guiding is also an important quality of scholarship. Indeed, frustration with the lack of action-guidance of much of political philosophy was an important impetus for the non-ideal turn in political philosophy about two decades ago. Yet without value-comprehensiveness, one cannot argue for a specific institutional design as a policy proposal. Policy proposals must aspire to be comprehensive regarding the values they consider, even if this will be hard, perhaps even impossible, to achieve fully.

This comprehensive value-pluralist method raises the question of how we know which values are relevant for a particular analysis. Ideally, the philosopher(s) conducting the analysis should be aware of all values that could *potentially* be relevant. The list of values should of course be open to revision, since we might have simply overlooked something and new values may become relevant due to social, technological, or other developments. An example of such a value is climate justice—it is currently a very important value, yet was not immediately relevant in the not-too-distant past.

Once we have a list of all potentially relevant values, we should ask the following question regarding each of them: what are the values at stake, at least in a significant way, for the problem, practice, or institution that we are analysing? Hence, for our current analysis, we should ask the following question: what are the values at stake with respect to inheritances and gifts and the taxation of them? Ideally, we should have a list of all relevant values and check them one by one for their relevance. It is possible to do this, but it would be very time-consuming. In practice, it is the philosophical method of engaging with existing arguments and with debates and critique with others that makes us come up with a list of relevant values. Existing discussions often trigger new thoughts, and these may point us to values that have erroneously been overlooked. Thus, the list of relevant values should always be *revisable*, given the risk of epistemic errors.

Based on my reading of the literature on the political philosophy of wealth transfers, inheritance, and bequests (and their taxation) and on engaging in debates with citizens about inheritance taxation for many years, I submit the following as the list of relevant values that should be considered when thinking about inheritance taxation: welfare, freedom, desert, equality of opportunity and social mobility, political equality and non-domination, and the values of family relationships and personal relationships. The fourth element of the Accessions Cap Proposal—the citizen's stake—could also be related to values of welfare, equality of opportunity, and the reduction of poverty, as Le Grand and Nissan (2003) have argued. This element is also related to another value, namely justice between age groups. This list of relevant values includes (albeit sometimes using slightly different names) the values that have been discussed in overview articles and books on inheritance taxation (e.g., De Maagt and Robeyns, 2017; Gosepath, 2023; Halliday, 2018; Pedersen, 2018; White, 2008).

Another aspect of comprehensive normative audit as a real-world political philosophy method is that it must specify which “real world” it is being applied to.^4^ That is, the feasibility constraints need to be spelled out. The first set of feasibility assumptions relates to the financial and institutional features that it is assumed exist. The analysis that will follow applies to a country that has a properly functioning fiscal infrastructure as well as a sufficiently high level of economic development. In such countries, we not only see significant economic inequalities but also high levels of average wealth among the richest, and thus children of parents in the top percentiles of the wealth distribution currently receive very large inheritances, while the bottom half of the population receives little to nothing. The second set of feasibility assumptions relates to attitudes that citizens and residents have towards inheritance taxation; their trust in the government's ability to spend those tax revenues well; and their trust in the government's ability to set up regulatory measures that can effectively prevent tax evasion. This will pose a challenge for anyone wanting to do real-world analysis when they are deciding which assumptions can still be considered “realistic.” On the one hand, these feasibility constraints are not immutable, as the fiscal capacities of a government can change over time and people's willingness to pay taxes can also change depending on the circumstances, including which ideology is dominant. On the other hand, being too optimistic about these feasibility constraints moves the analysis more into the direction of ideal theory, which real-world political philosophy wants to avoid.^5^ I will return to these challenges in the last sections of this paper.

Let us now turn to the analysis of the values that the scholarly literature as well as civic and political debates regard as relevant to the ethics of inheritances and gifts and the taxation of them.

## Welfare: The distribution of money

An analysis of how the design of inheritance and gifts taxation affects welfare should start from the most basic welfarist insight: *ceteris paribus*, the more egalitarian the distribution of money, the higher the corresponding welfare levels.^6^ This claim is based on the assumption concerning the decreasing marginal utility of consumption, whereby money is used as a proxy for consumption. A redistribution from a better-off person to a worse-off person will lead to a bigger welfare increase for the latter than the size of the welfare decrease for the former, and hence total and average welfare will increase. Thus, redistributive wealth transfer taxation, including progressive inheritance taxation and a wealth tax (on the holdings, not on the transfer), has been defended by economists on the ground that it could increase overall welfare, including by reducing poverty (e.g., Atkinson, 2015; Piketty, 2021).

What does this welfarist insight imply for the design of the institutions of inheritance and gift-giving and the taxation of them?

The Accessions Cap Proposal would give donors an incentive to give away their money to a larger number of recipients. Wealth would be more widely dispersed, which implies, *ceteris paribus*, higher overall welfare levels. Of course, the standard objection is the levelling-down objection, which is that due to the negative incentives caused by taxation, total production would be negatively affected, which would lead everyone to be worse off. The question of how the Accessions Cap Proposal would affect total welfare production will be discussed below.

A welfarist analysis would hold that the value of welfare or wellbeing will give us a *pro tanto* reason to have progressive taxation, including progressive inheritance and gift taxation. However, does it give us a reason to support the Accessions Cap Proposal, which imposes a limit on lifetime receipts of inheritances and gifts and thus includes a top marginal tax rate of 100%? To draw that conclusion, one must additionally accept either the claim that at some point the marginal utility of money approaches zero, or that although there is a welfare cost of imposing an accession cap, that cost is worth the benefits it generates.

I have argued elsewhere that the assumption that the marginal utility of money asymptotically approaches zero is plausible (Robeyns, 2017). At some point, having more money does not add to one's abilities to lead a flourishing life, since one's all needs have been met. However, this view requires a non-subjective account of wellbeing, e.g., based on the basic need theory or the capability approach.

If instead we use a preference-satisfaction theory of welfare or wellbeing, one might argue that the marginal utility will never become zero, since new preferences will always be created when one moves higher up the consumption ladder, and that we will never reach a situation in which all preferences that a person has are fulfilled. Someone who endorses such a subjective theory of welfare for institutional design could resort to the argument that the cost of imposing an inheritance cap, even if it lowers someone's preference satisfaction, can be justified given the gains of those with lower levels of preferences satisfaction *or* because of significant gains in other values, which will be discussed below.

The value of welfare taken alone would recommend institutions and policies that reduce inequalities in the post-transfer distribution of money. A gift and taxation scheme that would lead to a less inegalitarian distribution of money would, all other things being equal, be welfare-enhancing. The Accessions Cap Proposal does reduce inequalities—*a fortiori* because the tax revenue will be used for a citizen's stake, which should lead to a significant reduction in poverty and thus an increase in welfare (Le Grand and Nissan, 2003; Paine, 1796).

However, the analysis of the value of welfare immediately prompts the question of what the efficiency effects of accession taxation are. Is it not the case that the Accessions Cap Proposal is massively inefficient?

## Efficiency: Aggregate welfare and economic incentives

How does the Accessions Cap Proposal affect efficiency as standardly understood in the applied taxation-policy literature, that is, whether a policy change creates more aggregate welfare by giving people an incentive to enlarge the social product? Will the Accessions Cap Proposal lead to more economic activity or will it undermine economic activity and thus harm aggregate welfare levels?

The first question concerns the negative incentive effects on labor supply, which is a worry for all forms of taxation. As long as there is an additional person, the transferor cares about and to whom they would like to make a donation or leave an inheritance, the transferee is not taken beyond the accession limit and they will not be taxed. As long as those who currently have much more than the Accessions Cap are willing to spread out their wealth more widely, their transfers will not be confiscated. The well-off would thus continually have an incentive to keep earning (and thus working and producing). There would only be a very strong disincentive to work for already rich persons who only cared about donating to a very limited number of persons and who did not want to donate their additionally earned money to any person who was currently still below the accession limit. The Accessions Cap Proposal might, of course, drastically change how wealthy transferors think about donations and bequests, since they would be incentivized to think about a much wider range of potential transferees. This is not only because every person would have a cap on how much she could receive, but also because in comparison with many currently existing inheritance taxation structures, there would be no difference in the tax rates for children, grandchildren, other relatives, and those who are not family. The huge biases that current inheritance tax systems (with positive tax rates) entail against bequeathing to non-relatives would be removed, which would create a significant welfare-efficiency gain.

Another efficiency-based worry is that the Accessions Cap Proposal will lead to the destruction of companies. The objection, which is also raised against existing inheritances tax structures (Neuhäuser, 2023), states that it would be economically harmful if those who received parts of a company were to be (heavily) taxed. In many countries, family businesses are perceived to be the engine of the economy and employment. The worry is that the proposal could be fatal to those companies if they cannot simply be transferred to the next generation of business owners. This is also one of the motivations behind the large exemptions for family businesses in the inheritance tax regulations in some countries.

What should we think about those claims? It is true that if the limit of the Accessions Cap Proposal is low, this will not make it possible for the owner of a firm to hand over his firm to his favorite heir, and perhaps not even to all the heirs if the value of the firm is beyond the sum of the combined accession cap level of all the heirs.

Several responses disarm this objection (De Maagt and Robeyns, 2017; Haslett, 1997; Neuhäuser, 2023). The first response to this worry is that it should not be assumed that the children of current business owners are always suitable business successors. If one were to grant exemptions to the accession tax for inheritance involving a firm, the owner of the firm would have a strong incentive to give the firm to their children rather than to the person who would be best suited to running this business. Moreover, such an exemption could lead to a distortion in market competition, which tends to be accompanied by an efficiency loss in that particular market, as well as to unequal opportunities between entrepreneurs who are given a company versus entrepreneurs who build their business from scratch or have to buy one from another entrepreneur. The entrepreneur who was simply given his business under a fiscal regime of inheritance tax exemptions for firms has a better starting position that is not due to his efficient way of doing business. Moreover, granting tax exemptions for family firms provides very strong incentives for rich elderly people to incorporate their family wealth, which might create a significant welfare loss from tax avoidance of the richest.

Under which circumstances would the Accessions Cap Proposal harm the economy and thus score poorly regarding the value of efficiency? It would do so if the heirs to a business happen to be very good business successors and lose the possibility of continuing to operate the business. Therefore, the tax design should enable the children to continue the family business if that is what all parties involved prefer. This would not be difficult to realise: the accessions tax system could give heirs the right to buy a family company after the owner's death. The government could make this financially possible by providing long-term loans on favorable terms (Haslett, 1986: 138).

There are at least two reasons in addition to the efficiency consideration why such a possibility to continue the family business is important. The first reason relates to the value of the family. For many people, a family business not only has financial value; the company can be part of the identity of a family and the individuals that are part of the family (Neuhäuser, 2023). This proposal takes the value of the self-understanding of the family into account. Second, there is a risk that if the government simply sold family businesses to the highest bidder, many family firms would be swallowed up by private equity investors or other corporations, which would negatively affect the entrepreneurial ecosystem.

Given this amendment to the Accessions Cap Proposal, which should give heirs the option to continue the family business if they want to, we can conclude that the proposal will not negatively affect the efficiency of economic production.

## Freedom

The next value we need to consider is the individual freedom of the person who gives donations or, upon their death, will bequeath to their heirs. The view that individuals should be able to decide for themselves what to do with their wealth—consume it, donate it, or get rid of it in any other way that does not violate negative liberties—has a very strong appeal in the public realm (Halliday, 2023). Defenders of this right claim that any restriction of this freedom violates their fundamental rights.

Those who prioritize freedom typically either only care about the value of freedom or accord lexical priority to freedom in their weighing of different values when they are considering the design of fiscal institutions.^7^ Assuming the money earned is legitimately owned by a person, the freedom-prioritizing view then holds that this person should decide whether they give the money away during their lifetime or upon their death or spend it. Such an argument might be grounded in a particular theory. For example, Berrotarán (2022) has argued that the right to bequeath is a Rawlsian basic liberty and thus takes priority over concerns regarding equality of opportunity or the redistributive concerns embedded in the Rawlsian difference principle.

Yet if one engages in a real-world normative analysis, then this conclusion is premature, since we must also take the consequences for other values into account (Goodin, 1982). The methodological starting point of comprehensive normative audits is precisely that in a pluralistic society, citizens have reasons to care about a range of values, not just about freedom understood as negative liberty. There might be some citizens who only care about freedom or accord it lexical priority, but we cannot assume that all citizens have reasons to endorse this position.^8^

So what role should freedom play in the design of inheritance and gift taxation? Mill argues that there is a clash between the perspective of the donor and the recipient of the gift/inheritance. The donor should have the freedom to dispose of her own money that she has legitimately earned and can be said to deserve; the recipient has no claim to make at all. However, obviously, the freedom of the donor to give becomes meaningless if he can only give money to people who are not allowed to receive it. The value of freedom thus prompts us to design the institutions of inheritances and gift taxation in such a way that those who rightly possess money can donate in a way that does not negatively impact other values, or so that any potential negative impact on others is judged to be smaller than the gain in the value of being able to give more freely.

As I will argue in the next section, the negative effects of very large donations and inheritances on equality of opportunity and on political equality give us reasons to argue for a *limit* to the amount of wealth one can inherit or be given. The negative consequences of the transfer of wealth only apply to large transfers, so these consequences cannot be a reason against donations or inheritance of *all* sizes. This argument again provides a further reason to allow donations up to an accession cap for heirs and recipients. Donors can donate as much as they like, as long as they spread out their wealth among many recipients. Their freedom to give is thus not curtailed in terms of size—it is only qualified in such a way that it cannot negatively impact other values that donations affect.

## Desert

From the perspective of the donor, bequeathing can be seen as an exercise of their individual freedom. From the perspective of the recipient of an inheritance, however, an inheritance appears to be a clear case of undeserved wealth. There are different ideas about the precise interpretation and basis of desert, but what they have in common is that they refer to a certain characteristic or act of an individual. For example, you deserve an income because you have worked hard for it, or because you were willing to take financial risks by investing in a company. Yet, as has been pointed out by many scholars writing on the ethics and legislation of inheritance and gifts, heirs have not worked for an inheritance, and it is purely coincidental which family they were born into (Duff, 1993; Gosepath, 2023; Haslett, 1986; 1997; Mill, 1848). If desert is the only value that matters, inheritances should be abolished completely unless all inheritances are exactly the same size.

However, what if an inheritance has been conceived by the inheritor, or is perceived by the heir, as *a reward*? For example, this might be argued to be the case when a child has performed a lot of unpaid work on their parents’ farm, which they hope to inherit later. Or when someone is taking care of a frail elderly person, or doing so to a much larger extent than their siblings. Elderly care can be very burdensome and often comes with significant costs to those who provide the care, not just in terms of time requirements and forgone earnings, but also with respect to various other aspects of the quality of life of the carers (Den Hartog, 2018; Philp, 2016; Stark, 2005). In this case, one might argue that the heir is, in a moral sense, deserving of the inheritance. Moreover, Hacker (2023) has argued that inheritance taxation should be reformed to allow for such rewards for providing elderly care by means of will contracts, since this would provide elderly people with an important means of ensuring that they are cared for in their own homes.

However, it does not follow that the only possible, or even the most appropriate, way to recognize this work is by donating money (during one's life or upon death) to the caregiver. There are at least four other ways to think about this question. The first is to socialize the cost of caregiving, either by giving caregivers paid care leave or by giving older people access to good-quality elderly care that is provided by the state free of charge (or cheaply). The second is to see elderly care as a matter of reciprocity over a lifetime. Take the case of an adult child who cares for their frail old parent. Why should she be entitled to a financial reward if the parent has done a lot of unpaid (care) work for the child when they were younger? The problem with this second approach is that when there are multiple children and an unequal division of elderly care work, the reciprocity is very uneven. Another problem is that all children receive care when they are young, but not all adult children have to provide care for their elderly parents; the burdens of providing elderly care are spread very unevenly among adult children, simply because some elderly people die before they need care and others need intensive care for many years. The third approach is to recognize the work for what it is—work—and pay a wage. If a frail elderly parent has three adult children and one of them cannot have a paid job due to the full-time care they provide for this parent, it seems fair that this person is paid to do this work, which would also be compensation for forgone opportunities. If one looks at this purely from an economic point of view, one would argue that this work should be paid for *when the work is done*. Fiscal fairness requires that the recipient of this non-market wage should pay income taxation on it, since there is from a fiscal point of view no relevant distinction between a family member being paid for these services and an income that is earned by elderly care workers who offer their services on the market.^9^ A fourth approach would be to increase the lifetime accessions cap of family members who have spent time providing unpaid care work by a fixed amount relative to the time the care was provided for. An additional advantage of this last approach is that it would allow people other than the person receiving the elderly care to contribute to the reward/compensation, including other relatives who also have prima facie a moral duty to provide that care.

There are clear normative differences between these four approaches to addressing the claim that inheritances might be seen as a reward for care work, but none of them would require us to abandon the Accessions Cap Proposal.

## Equality of opportunity and social mobility

Another value that is important in the ethics of taxation is equality of opportunity. The argument here is that permitting bequests and donations reproduces inequalities of wealth. In a world without bequests and donations, there would still be inequality of wealth, but the inequality would be much smaller than the large wealth inequalities that have been documented in recent years. At present, many citizens receive nothing to a mere few thousand dollars over their lifetime, while others receive millions or indeed billions, just because they were born to different parents.

Various accounts of equality of opportunity are proposed in contemporary political philosophy, yet to the extent that these theories are applicable to the real world, they would all hold that wealth inequalities that are generated through (large) inheritances and gifts undermine equality of opportunity. There are multiple ways in which inequality in wealth undermines equality of opportunity (De Maagt and Robeyns, 2017; Gosepath, 2023; White, 2008). First, money simply creates (more) opportunities, and as Chambers (2009) has argued, inequalities in outcomes are the sources of inequalities in opportunity. The distinction between outcomes and opportunities that is based on a time-slice analysis does not survive a conceptualization that takes the dynamical interactions between the two into account. Second, inequalities in wealth are the cause of several aspects of ill-being, such as differences in physical and mental health, obesity, violence and crime, teen pregnancies, and weaker social cohesion in neighborhoods (Rowlingson, 2011; Wilkinson and Pickett, 2009).

Many recipients of an inheritance are in their fifties, and thus one might wonder to what extent inheritances really undermine equality of opportunity, since recipients are already in the second half of their lives. Yet as Wolff (2020) rightly argues, inequality in inheritances leads to a high level of inequality of opportunity for good ageing among pensioners, with some living in poverty or with very modest means, whereas others can enjoy their old age without any housing costs (i.e., mortgage-free and with no rent to pay) and therefore have more money to spend on leisure activities. Moreover, knowing that one might receive an inheritance later in life works as an insurance cushion earlier in life, allowing one to take loans or more risk. In addition, official data for the US show that while gifts are most often received when recipients are in their twenties, those in the top 10% of the income distribution are much more likely to receive gifts than other people and the size of those gifts is significant (more than 250,000 dollars) and more than three times what other recipients receive on average.^10^ Not much of an explanation is needed here to illustrate that this creates inequalities of opportunities across the lifecycle.

Daniel Halliday (2018: 127) gives a further argument about how wealth inequality indirectly affects equality of opportunity by highlighting how it affects multiple generations. According to Halliday, the normatively most salient problem with inheritance is the cumulative impact of these wealth transfers on the ability of wealthy groups in society to create better opportunities for their children. It thereby cements social stratification and reduces social mobility. Some might counterargue that an inheritance is often received too late in life to make a difference to the social position of its immediate beneficiary. However, Halliday convincingly argues that inheritances do impact the ability that the immediate beneficiary has to create better opportunities for *their* children, for instance by having enough money to pay for expensive sports, musical instruments, or private tutors. Or by simply being part of the social networks which create better opportunities in life and which are typically, or at least to a significant extent, related to one's economic position. Dynastic wealth, that is, wealth that is passed on from generation to generation, thus adds a further layer to undermining equality of opportunity. If the cap of the Accessions Cap Proposal were sufficiently low, it would also impose a clear cap on dynastic wealth creation, in addition to advancing equality of opportunity by introducing a citizen's stake.

If equality of opportunity were the only value that mattered, the corresponding institutional design would be the complete abolishment of inheritances and gifts of any significant size. Inheritances and gifts would then have to be fully taxed, and the tax revenues used in a manner that aimed to advance the goal of equal opportunities for all.

## Political equality and non-domination

There is a particular form of equal opportunity that requires a separate discussion: the opportunity to influence politics. The concentration of vast amounts of wealth also has effects on the functioning of democracies, because a democracy entails citizens having equal opportunities to exercise political influence. However, if inequality of wealth becomes too large, mechanisms may arise that undermine political equality between citizens (Christiano, 2012; Rawls, 1999: 245). For example, citizens with large fortunes have more opportunities to lobby the government to develop specific policies, including policies that allow them to pay less tax than the middle classes. Wealthy citizens can also use their media enterprises to filter news coverage and influence discussions in the public sphere. Or they can spend much more on electoral campaigns, which leads to elected politicians who cater more to their interests than to those with less money.

One might object that the risk of undermining political equality is not very large in countries in which using financial means to influence politics is mainly done by businesses.^11^ Yet even in countries where donations by businesses are largely restricted, such as in countries that are part of the European Union, we still see that individuals differ vastly in their ability to turn money into political influence. And if inheritances and gifts are not taxed, it implies that the beneficiaries of those gifts and bequests not only inherit money but also the opportunity to influence politics. How much money others receive is thus a matter of importance for all in a democracy.

For republican thinkers, freedom as non-domination is the core value. While the need for a minimum level of financial resources to protect republican freedom has long been recognized, more recently it has also been argued that republican freedom requires a threshold for how much wealth a person can hold relative to other persons (Dumitru, 2020; Icardi, 2023). The values of political equality and non-domination would thus be enhanced by having a cap on how much one could receive in terms of inheritances and donations and by having wealth spread out more evenly in the population.

There are two different normative positions one could take here. One is to say that all inequalities of political power should be eliminated. If one endorses this view, wealth concentration should be avoided independent of whether that wealth is based on inheritances and gifts or has been created in one's own lifetime. The other, weaker, view is that what is especially troubling is *hereditary* political power. According to Fleischer (2016), this view is at the very basis of the self-understanding of the citizens of the USA. She argues that if we want to minimize dynastic wealth transfers which lead to the inheritance of social and economic power, the best tax instrument is a lifetime progressive accessions tax. However, Fleischer's proposed design has two features that are radically different from my proposal. The first is that her proposal is structured similarly to the moderate interpretation of Mill's proposal—hence it has a lifetime accessions *exemption* with a progressive tax rate above that level. Under this design, a large enough inheritance could still make one into a billionaire, which seems strongly at odds with values of political equality and non-domination. Second, Fleischer claims that the exemption limit should be somewhere between 10 and 20 million dollars, since she takes that to be the level when wealth starts to translate into political power. She does not provide empirical evidence for that assumption, but one might wonder whether that limit should not be much lower.

## The value of the family

A value that is often invoked to defend the right to bequeath and donate, and derivatively to receive, is the value of the family. It is often believed that parents have special duties towards their children and that this requires allowing them the freedom to donate or bequeath to their children. But is this reasoning correct?

In order to answer that question, we must first be clear about what the value of the family precisely entails. Harry Brighouse and Adam Swift (2009, 2016) have analyzed what, if anything, makes the relationship between parents and children so special and what moral and political rights follow. They argue that the family is an important social institution because it allows some valuable things which cannot be realized through other social institutions—what they call *familial relationship goods*. Specifically, they argue that the parent–child relationship is qualitatively unique, for at least two reasons. First, the parent is a unique advocate of the child's interests. Second, they can also develop a unique relationship with the child through the special form of intimacy that this relationship makes possible. Society should therefore support these family relationships, for instance by enabling parents and children to spend sufficient time with each other.

What does this mean for inheritances and inheritance taxes? Brighouse and Swift argue that inheritances undermine equal opportunities and that the competitive advantage that parents can give to their children through an inheritance cannot be justified based on the value of familial relationship goods. It is true, though, that certain specific goods, such as a house that has been in the family for generations and that is the place of many important memories, can play a role in the value of family relationships, and therefore should be allowed to be passed on to the next generation of the family. Brighouse and Swift thus conclude that almost all forms of inheritance are not necessary for the existence of what is intrinsically valuable to the parent–child relationship, and that inheritance, moreover, leads to competitive advantages for those who inherit (a lot) and thereby undermines the ideal of equal opportunities, which they highlight as another important value.

However, this might not be all there is to be said when we are looking at inheritances and gifts from the perspective of family ties and personal relationships. Neves (2023) has argued that inheritance transfers foster an important sense of belonging in families that is based on the norm of intergenerational reciprocity. Family members have benefitted from material support, including gifts and inheritances, provided by previous generations and want to repay this duty by passing on an inheritance to their own children. Schmidt am Busch (2023) has argued that bequests made through wills help us to remember people and to sustain memories of people we were close to. This is at least as much the case for donations made while the donor is still alive.

The design of the institution of accessions taxation should take family values into account, including considerations of intergenerational reciprocity, because for many people it is an indispensable part of how they live family life. For many people, memories of personal (especially ancestral) ties are important and can plausibly be understood as part of how they conceive themselves as human beings over generations, and this aspect should also be considered in the design. However, it does not follow that any and all transactions should therefore be exempted from taxation, as Neves points out. Imposing a lifetime accession cap should provide an expressive space in which the person who is giving or bequeathing can protect the value of personal ties and contribute to the remembrance of the deceased. The details of the institutional design should make sure that goods that are specific to the family (e.g., the family house) can remain in the family by having similar policies that allow the intergenerational intra-family transmission of family firms.

If the argument that, bracketing special needs and disabilities, caring about one's children is not a reason to be morally permitted to give them gifts and bequests, how can we explain that almost all parents have the intuition that they are doing something morally permissible if they leave their children money after their death? Perhaps most parents regard the duty to care for their child and defend their child's interests as a core aspect of parenthood, and as legitimate. As long as they are alive, parents can try in various ways to help their children who get into trouble financially. If parents are deceased, there is very little that they can still do—except to make money available which can prevent their (adult) children from becoming destitute or to enable them to use those resources to solve their problems. Having some assets is an insurance against possible misfortunes, and parents may be aiming to prevent their adult children from suffering from such misfortunes rather than trying to secure an unfair advantage for their children. And in the non-ideal world we live in, in which the welfare state has crumbled in many places and we cannot insure ourselves against all forms of bad luck individually or socially, parents hope that they can mean something to their children after their death. The Accessions Cap Proposal would allow parents to express their parental love through gifts, but only up to a certain level, since inheritances and gifts are not only buffers against misfortune but also possible engines of unfair advantages.

## Feasibility: Organizing democratic support

Several of the values we have analyzed so far point towards strongly restricting the right to bequeath and thus to inherit. Yet we know from the literature that inheritance taxation is one of the most unpopular of all taxations (Beckert, 2008; Prabhakar, 2015). Even after discussions of all arguments for and against in deliberative workshops, there are more participants who oppose inheritance taxation than support it (Lewis and White, 2006).

One hypothesis is that such opposition might be due to a lack of trust in the government, and that knowing how the revenues from inheritance taxation would benefit citizens could increase support for it. Lewis and White (2006) conducted two deliberative workshops on inheritance tax and found that hypothecation of the inheritance tax revenue did indeed increase support for this tax. A recent Dutch survey study shed some further light on these issues. Respondents overwhelmingly *rejected* the idea of a limit to what one could receive from inheritances. Yet in the same study, 69% of respondents answered “agree” or “strongly agree” to the statement that if the government had to choose between reducing the public provisions for the most vulnerable citizens or raising taxation on the rich and the superrich, they had to opt for the latter (Robeyns et al., 2021: 126). This confirms suggestions made by Lewis and White (2006) that if the issue is framed in terms of decreasing inequalities while at the same time making clear what is being done with the taxation revenue, the support base for increased taxation is much bigger.

The Accessions Cap Proposal would gain much in terms of political feasibility if it also had a component that spells out what will happen to the tax revenues. This would be strongest in the case of hypothecation—the earmarking of specific tax revenues for specific policies (Halliday, 2018: 204–206).

This could be done in different ways, and providing details concerning how the tax revenue will be spent creates a further opportunity to increase the total positive consequences of the proposal. After all, one could redistribute the raised funds on a precise per capita basis to each citizen. This might increase the political feasibility, but it would not be much of a gain in terms of value. Instead, one might choose to use the funds that have been raised to reduce inequalities between age groups, in particular the precarious position of young adults. As Bidadanure (2021) has argued, young adults are in several ways put in a structurally weaker position than older adults. They have less money for political donations and hence weaker political power; they also have less money they could use to turn into opportunities and are often juggling multiple responsibilities at once. Yet we also know that not all young adults are equally (dis)advantaged. Some of them have already received large gifts from their parents or know they will inherit money later and use this knowledge to take out loans without the feeling that there is any risk in doing so. Redistributing the revenues that have been raised via the accessions tax and turning them into a stake for young adults would thus reduce inequalities between age groups and further enhance equality of opportunity between young adults who happened to be born in different social classes. It is beyond the scope of this paper to argue what exact form that stake should take—for example, whether it should be basic capital or a basic income, at what age it should be received, and so forth. Several proposals have been made in the literature on stakeholding, and multiple institutional designs have been suggested there (e.g., Ackerman and Alstott, 1999; Atkinson, 2015; Dowding et al., 2003; Piketty, 2021). The point to stress here is that the choice between those different designs should not only meet the requirement of securing democratic support for the complete Accessions Cap Proposal, but should also be used as an opportunity to create additional value, for example, by addressing inequalities between the young and old.

## Weighing all values

We are now able to conclude the analysis and bring the *pro tanto* arguments based on the different values together.

If equality of opportunity or desert were the only values that mattered, the corresponding institutional design would be the abolishment of inheritances and gifts of any significant size. Inheritances and gifts would then have to be fully taxed, and the tax revenues used in a manner that would maximize equal opportunities for all. Yet a central premise of the normative audit is that there are a variety of values at stake in a just design of taxation rules, and hence any proposal for a specific taxation design must weigh these different values. Abolishing inheritances and significant gifts completely would violate the freedom of the donor and negatively affect the value of efficiency, thereby possibly lowering overall welfare levels.

The analysis of the above values has shown that in the design of a scheme for inheritance taxation several values are at stake, which has implications for that design. First, it is hard to defend having no inheritance tax at all, which is currently the case in countries such as Australia, Austria, China, New Zealand, Norway, and Sweden, and de facto to a very large extent in the USA, where the estate tax only applies to bequests over 12.92 million US dollars for an individual (Fleischer, 2022: 91). Inheritances are undeserved, and given the large inequalities in inheritances and gifts, they undermine equality of opportunity and solidify social stratification. Anyone who cares about welfare or wellbeing as a public value should also favor implementing taxation on very large gifts and inheritances, since total and average welfare and wellbeing increase with the wider dispersal of money. The only way in which we could justify having no inheritance tax at all is to deny the importance of all these values, which seems a rather radical position which even classical liberals would not endorse (given their commitment to equality of opportunity). The only people who might endorse this view are those who only care about individual (negative) freedoms. Inheritances and gifts thus have to be taxed; the question is what the design of that taxation should look like. The weight one attaches to the different values and exactly how one understands those values, as well as some empirical information that is relevant for the relationship between those values and the design of inheritance taxation, will determine which design is compatible with a particular set and weighing of values.

Recall the specific proposal I would like to defend:

*The Accessions Cap Proposal:* the government imposes a lifetime limit on the financial value of what each person can receive in terms of inheritance and gifts. Once that limit is reached, the government implements a 100% tax rate on any further inheritances or gifts received. The tax revenue thus generated is used to finance a citizen's stake.

This proposal limits the total value of lifetime inheritance and gifts that a person can receive to a level which tries to balance the following desiderata: first, not be so low that it would still give donors a significant freedom of choice between spending their earned money or passing it onto others; second, protect equality of opportunity and the value of political equality, which are jeopardized by inheritance and gifts with larger values, and third, enhance the value of wellbeing directly due to the incentives the proposal gives to spread one's wealth among more recipients. With suitable complementary policies to allow family firms to be acquired by heirs, it also does not score badly regarding the value of efficiency.

Another reason why one should be allowed to receive, through gifts and inheritances, at least some limited amount, is that this allows for the caring attitudes and the wish to meet norms of intergenerational reciprocity that explain the motives of parents who want to bequeath to their children. Given that the government cannot be an insurer for all bad luck in life, and given that having some wealth works as insurance against the stress and anxieties that the risk of misfortunes can cause, our societal institutions should encourage people to care for others—including in a financial sense. This should only, however, be encouraged to the extent that it does not crowd out the social insurance function of the government, since that function is not only justified based on considerations of fairness but also on considerations of efficiency (Heath, 2006). There should not be a privileged status for children in terms of lower tax rates or higher levels of exemptions, though, because this caring attitude need not only exist between parents and children. Taking away the privileged positions of children over other heirs or transferees in the inheritance and gift tax systems would be a major difference to current regulations in most countries that still have inheritance, bequest, or gift taxation.

Clearly there are many desiderata, and they point to different upper limits on accessions. So which one should we prioritize? Citizens will probably have different legitimate views here, which will depend on how they weigh these different desiderata. I believe that the main factor that should determine where to put the limit would be the level of inheritance and gifts that would be the pivotal point at which it would start to significantly undermine equality of opportunity and political equality. I would consider this weighty enough to limit lifetime inheritance and gifts, but they do not give us a clear answer regarding where the limit should be put. This depends, in part, on whether there are laws in force in the relevant jurisdiction that protect the political sphere from the sphere of money, such as campaign funding regulations. Since equality of opportunity is a value with a very wide reach, it also depends on social and economic policies that affect it, such as policies that deliver high-quality social housing, education, and health care. If these are guaranteed, the additional opportunities that can be bought with modest levels of gifts and inheritances are limited. However, vast fortunes can still buy opportunities not available to the vast majority of citizens and which even a decent social state will not be able to fund for all. The goal of the social state should be to secure minimal thresholds of meeting basic needs and creating opportunities for all; the goal of the Accessions Cap Proposal should be to minimize the probability that these accessions will create access to exclusive opportunities not available to others, including access to disproportionate political influence. Although we need empirical research to find out what the accessions cap should be, if I were to be forced to give a number, I would say my ballpark number is 100,000 US dollars. This is an amount that protects all values that have been put forward in defence of inheritances and gifts, yet it is also an amount that does not significantly harm equality of opportunity and does not have, I would assume, any influence on political equality.^12^ No doubt a range of other questions regarding implementation arise, but discussion of those would require another paper.

## Lessons for real-world political philosophy

In closing, I want to make three final comments that relate to the degree of realism of the Accessions Cap Proposal. To what extent is this really a proposal for the world as it is? And what do the arguments in this paper tell us about the attempts to develop “real-world political philosophy” in practice?

Wolff (2020) believes that real-world political philosophy concerning inheritance taxation should be specific to a country and should consider historical contexts. Wolff argues that in the UK, the most important progress can be made by limiting exemptions in the bequest tax code, as they currently lead to many heirs paying no taxation on bequests at all. Yet while one may agree that this would be the first thing a political philosopher would advise a politician to do, surely real-world political philosophy should be more ambitious than only point at aspects of the current legal and social design that facilitate tax evasion and avoidance. Real-world political philosophy should also aspire to argue in favor of institutional designs that might be genuinely different from the current ones and that can be aspirational for many countries. Yet this does require the further development of methods for creating such institutional designs, especially with respect to taking different types and different degrees of feasibility constraints into account.

One particular feasibility-based objection to the Accessions Cap Proposal relates to international capital mobility. Is the Accessions Cap Proposal feasible in a world in which the richest can shift their wealth to territories without inheritances and gift taxation or store it in fiscal paradises? This is an objection that holds for any kind of taxation on wealth or profits (or other tax grounds that are moveable across jurisdictions) but also holds *a fortiori* if a tax scheme includes a marginal tax rate of 100%. If a country were to implement a strict limit on what one can receive in the form of gifts and inheritance, then surely many households with wealth above that level would move, or move their wealth, to a jurisdiction with different rules. The solution here is the same as for other measures that limit the wealth and power of millionaires and billionaires: we should first work on realizing the *preconditions* that make the implementation of progressive redistributive taxation schemes feasible, e.g., by closing tax havens and establishing an international regulatory framework which is overseen by an international tax authority. Recent book-length arguments and proposals make clear that *it is technically possible* to drastically limit tax avoidance and evasion (Dietsch, 2015; Saez and Zucman, 2020). It is not simply “unfeasible” in the sense that there is a nature-made form of unfeasibility applicable here, and it is not technologically unfeasible either. What it requires is the political will and the political power of those who want to change this—and then their concrete actions. However, this objection does show that the Accession Cap Proposal is “less realistic” than, say, Wolff's proposal to eliminate the exemptions in the current gifts and inheritances tax code in the UK.

Another question about the degree of realism of the Accessions Cap Proposal is raised by the question of to what extent it takes into account the existing failures of the state to create or sustain social institutions that address socio-economic injustices. If the state fails in its role as the primary agent of justice, citizens with financial means might want to use those means to meet their obligations as secondary agents of justice. An example of this is the case of parents of children with significant special needs. If parents have a child for whom they are the primary responsible adults and that child cannot (sufficiently) take care of themselves after their death, this may be a reason to justify an inheritance, because the state may not fulfil its duty to meet the needs of that person with special needs. Both Mill and Haslett would agree, since they argue that parents should be able to leave bequests to their child until that child can provide for themselves.

This example illustrates that real-world institutional analysis has to take into account any existing injustices that are caused by the state not meeting its moral obligations. In the vast majority of countries at the time of writing, we have a long way to go before we have a situation in which the state meets the needs of vulnerable people. In our world, it might be argued that parents of vulnerable children (such as physically or cognitively disabled children, or children with addictions, disorders, or other psychiatric problems) should have the right to keep taking care of their children beyond their death by leaving them an inheritance up to the size of what is needed for meeting their basic needs. After all, it would not be about trying to obtain a competitive advantage for their child, but only about ensuring that their vulnerable child will lead a minimal good life in a society that doesn’t sufficiently care. Moreover, most disabled people already have much more constrained opportunities in life. We thus have a reason, if a state fails to meet its duties as the primary agent of justice, to allow donations to a fund that will allow vulnerable, disabled citizens to lead good lives.^13^ The precise design of the fiscal regulation of such a trust fund is beyond the scope of this paper, but it will be important to make sure that such trusts cannot be abused, as this would again create more opportunities for tax evasion by those who can afford those fiscal routes.^14^

## Notes

1. The Accessions Cap Proposal applies to natural persons receiving gifts and inheritances, not to charities. Discussing the most desirable design for the gifts and inheritance rules that apply to charitable organizations is beyond the scope of this paper, but the analysis provided in this paper does not give us a reason to include a cap in that design. However, this is not to say that charities are not crucially important for a real-world analysis of inheritance and gift taxation. Quite the contrary, since the very wealthy use charities as personal wealth shelters, sometimes also employing their children in them, thereby in effect bequeathing what would otherwise be taxed (Reich, 2018).

2. I modify here my earlier statement of this method (Robeyns, 2020).

3. I will use “value” in this section as shorthand for “values and other normative goals,” since not all goals that citizens find important are values in the philosophical sense.

4. I thank an anonymous referee for pressing me to develop this point more explicitly.

5. The question of how to deal with different types of feasibility in real-world methods requires further analysis that goes beyond the scope of this paper. Although this might be seen as a weakness of the present paper, I suggest that the analysis in this paper can be used as a case study that should lead to the further development of this method. I intend to take this up in future work.

6. I make no distinction here between “welfare” and “wellbeing,” given that there are no agreed-upon common definitions of those terms in the relevant literature, which spreads over at least four disciplines (economics, law, philosophy, and politics). Moreover, any distinction one might endorse is not relevant to the arguments provided.

7. Some libertarians endorse the freedom-prioritizing view, but other libertarians endorse a pro-market view. And since the freedom to make a bequest is not a market freedom, they might not object to inheritance and bequest taxation. For the different views that fall under the label of “libertarianism” and their diverse positions on the taxation of inheritances, bequests, and gifts, see Melkevik (2019) and Fleischer (2022). I thank an anonymous referee for pressing me to clarify this.

8. Moreover, Halliday (2023) has argued that it is not at all obvious that the idea of a property right itself entails an unrestricted and unqualified freedom to make a bequest or transfer.

9. Hacker (2023: 152) argues that the state should refrain from taxing the amount that carers will receive as an inheritance as if it were income because the state is enriched by saving the cost of publicly funding the care. However, that claim does not necessarily hold. An alternative institutional design is that the state provides all elderly people who need care with the same financial support (possibly applying means-testing), which they can use to pay for their care whether they live in a care home or have carers visit them in their own homes.

10. https://www.federalreserve.gov/econres/notes/feds-notes/how-does-intergenerational-wealth-transmission-affect-wealth-concentration-20180601.html.

11. I thank an anonymous referee for raising this objection.

12. Note that this is still much higher than the value of the median inheritance in many countries. In the USA, one of the richest countries in the world, 90% of the population will receive an inheritance of less than 60,000 US dollars (many receive nothing). The top 1% expects an inheritance of on average about 940,000 dollars; the next 9% 266,000 USD. See https://www.federalreserve.gov/econres/notes/feds-notes/wealth-and-income-concentration-in-the-scf-20200928.html, table B, panel B. Thus, putting an accession cap at 100,000 US dollars would not affect the inheritances of 90% of the population but would significantly reduce the negative effects that the vast inheritances received by the top 10%, especially the top 1%, have on important public values.

13. Note that the same reasoning applies to donations to funds or persons for purposes that amount to failures of the state to provide what citizens are entitled to based on human rights and in many countries also constitutionally entitled to but which the state fails to secure. For example, donations to cover legal fees which are needed to secure access to justice, or donations for medical bills for people without (adequate) health care insurance in the USA.

14. There will be other objections that might require further analysis. One question is whether the Accesssion Cap Proposal would also be applicable to Indigenous communities, for whom land is often not a resource akin to an element of wealth, and for whom the proposal raises questions of how it would affect community self-determination. Another objection is that one might worry that most parents do not like to talk about their inheritance with their children, yet they need to know whether their children have already received donations and inheritances up to the level of the cap's limit. One solution to this problem would be to allow donors to provide a ranked list of people to whom their donations should be given. If someone has already received up to their limit, the inheritance will be offered to the next-listed person, and so forth. I thank Jeremy Webber and Huub Brouwer for raising those objections.
